# Mortality and priority level for ICU admission in the setting of limited critical care beds in El Salvador

**DOI:** 10.1186/cc10991

**Published:** 2012-03-20

**Authors:** V Segura, NR Reyes, ME Tejada, EM Zolano

**Affiliations:** 1 Hospital San Rafael, Santa Tecla, El Salvador

## Introduction

In El Salvador there are a limited number of ICU beds. The ICU bed per inhabitant ratio is only 0.7 per 100,000 in a country with a population of 6,071,774 [1]. The aim of this study was to show the impact that the ICU bed deficit has on the mortality of the patients admitted to the internal medicine floor.

## Methods

We conducted a descriptive, cross-sectional study. A nonprobabilistic sample was estimated using EPIDAT 4.0 (mortality rate 16%, 95% CI, *P *< 0.05). We enrolled 513 patients admitted to the Internal Medicine ward, from June to November 2011. All patients were evaluated using the ICU admission priority criteria of the Society of Critical Care Medicine (SCCM). We divide the patients into high priority (SCCM priority levels 1 and 2) and low priority (SCCM priority levels 3 and 4) for ICU admission. The probability of death using APACHE II score and mortality rate was calculated for each group, in order to obtain the Standardized Mortality Ratio (SMR). A *t *test and a Mantel-Haenszel test were used for statistical analysis between groups.

## Results

**Figure 1:**
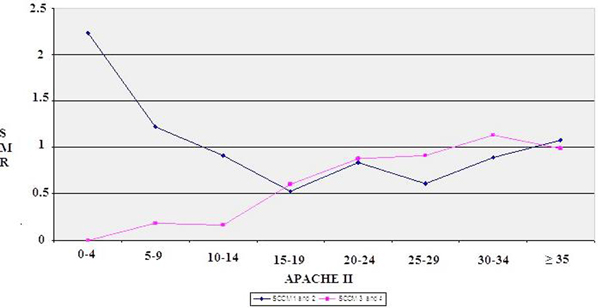
**SMR according to APACHE II and SCCM criteria**.

A total of 513 patients were included in the study; 101 patients in the high priority group and 412 patients in the low priority group. There was a significantly higher mortality (*P *= 0.048) in the high priority level group especially with an APACHE score less than 9.0 (Figure 1).

## Conclusion

The study shows that there is an increased mortality rate in patients with high priority level for admission to the ICU with an APACHE II score less than 9 points. This represents 90 patients/year whose survival and prognosis could be improved by increasing the number of ICU beds available.
